# Ovariotomy

**Published:** 1868-12-15

**Authors:** 


### *Ovariotomy.*

Dr. Dunlap, of Springfield, Ohio, has performed ovariotomy on 36 patients, since 1843. Of these, 13 were unmarried. The operations were all by the long incision, and only two were without anæsthetics. Nine died after operation; one from peritonitis, two from hæmorrhage, one from chloroform, one from accidental overdose of morphine, one complicated with cancer, one from exhaustion, one from congestion of the brain, and the ninth from excessive vomiting. Three of the successful cases have died since their recovery from the operation, of other diseases; the remainder are all now living, and in good health.—*Boston Med. Jour.*

